# Hydrodynamic Interaction Between Tear Film and Air Puff From Noncontact Tonometry

**DOI:** 10.1167/tvst.11.2.2

**Published:** 2022-02-01

**Authors:** Atieh Yousefi, Yanhui Ma, Cynthia J. Roberts, Sayoko E. Moroi, Matthew A. Reilly

**Affiliations:** 1 Department of Ophthalmology & Visual Sciences, The Ohio State University College of Medicine, Columbus, OH, USA; 2 Department of Biomedical Engineering, The Ohio State University, Columbus, OH, USA

**Keywords:** noncontact tonometry, aerosolization, droplet formation, biomechanics, tear film, air puff deformation testing

## Abstract

**Purpose:**

The purpose of this study was to investigate the mechanism of potential droplet formation in response to air puff deformation with two noncontact tonometers (NCTs).

**Methods:**

Twenty healthy volunteers were examined using two NCTs, Ocular Response Analyzer and Corvis ST, and two contact tonometers, iCare and Tono-Pen. High-speed videos of the tear film response were captured with at spatial resolution of 20 microns/pixel at 2400 fps. Droplet size, droplet velocity, distance between air puff impact location, and the tear meniscus-lid margin were characterized.

**Results:**

One subject was excluded due to technical issues. Droplets were detected only in tests with instilled eye drop. Videos showed the tear film rolls away from the apex while remaining adherent to the ocular surface due to the tendency of the fluid to remain attached to a solid surface explained by the Coanda effect. Twelve out of 38 videos with an eye drop administration showed droplet formation. Only one resulted in droplets with predominantly forward motion, which had the shortest distance between air puff impact location and lower meniscus. This distance on average was 5.9 ± 1.1 mm. The average droplet size was 500 ± 200 µm.

**Conclusions:**

Results indicate no droplet formation under typical clinical setting. Hence, standard clinical use of NCT tests is not expected to cause droplets. NCT testing with eye drop administration showed droplet formation at the inferior eyelid boundary, which acts as a barrier and interrupts tear flow.

**Translational Relevance:**

Study of tear film interaction with NCT air puff shows that these tonometers are not expected to cause droplet formation in standard use and that if external drops are required, both eyelids should be held if patients need assistance to maintain open eyes to avoid droplets with predominantly forward motion.

## Introduction

Since the outbreak of a novel coronavirus, severe acute respiratory syndrome-coronavirus 2 (SARS-CoV-2), and the worldwide coronavirus disease 2019 (COVID-19) pandemic, many clinical practices saw an urgent need to re-evaluate the safety of their techniques and instruments to assure the safety of both patients and medical staff. Despite COVID-19 being primarily a respiratory disease, studies have identified multiple ocular symptoms, including conjunctivitis,^1^^–^^5^ increased ocular secretions,^1^^,^^5^^–^^7^ ocular pain,^6^^,^^7^ and dry eye.^1^ There is less evidence supporting disease transmission through tears or ocular tissue. Xia et al. demonstrated the presence of SARS-COV-2 by real-time polymerase chain reaction (RT-PCR) from ocular samples from patients with conjunctivitis and argued that although the prevalence of virus in tears is low, the disease transmission via the eyes is still possible.^8^ Liang et al. and Wu et al. replicated these initial results.^7^^,^^9^ Ho et al. have reviewed 20 case series and case studies comprised of 2228 patients diagnosed with COVID-19, where 4.3% of subjects manifested ocular symptoms and only 0.5% had positive viral nucleic acid in ocular swabs.^10^

Given this speculative evidence that ocular fluids may be an infection route or serving as a source of disease spread, it is critical to identify sources of potential disease spread via common diagnostic devices, especially during this COVID-19 pandemic. Noncontact tonometers (NCTs) use an air puff to deform the corneal surface and assesses intraocular pressure (IOP), as well as characterize ocular biomechanics with respect to the air-cornea interaction. Recent studies have suggested the potential for aerosolization of the tear film with NCTs which would pose a risk with the clinical use of these devices during this pandemic with concern of possible viral load in the tears.^11^^–^^13^ However, the instructions for caution have been controversial and other research groups have contradicted such findings, indicating that NCTs do not generate droplets unless eye drops are given prior to the examination.^14^^,^^15^ Given the recent emergence of several COVID-19 mutations, which reportedly have a higher infectious rate, as well as increasing evidence that the major disease transmission route is airborne, it is timely to characterize the potential for droplet formation with these devices. In this study, we conducted high-speed imaging of the NCT air puffs impacting the corneal surface in vivo during a standard examination. The goals of this study are to address the question of tear droplet formation and to provide insight into the mechanism of tear film displacement.

## Methods

### Study Design and Subjects

To assess the interaction of air puff deformation on tear film dynamics, 20 healthy volunteers were recruited. The study was approved by Institutional Review Board (IRB) of The Ohio State University. Informed consent was obtained from the subjects after explanation of the nature and possible consequences of the study. The exclusion criteria were clinical history of dry eye or tear film instability. Testing was conducted in the right eyes only.

The robust study design was intentional using two different NCTs, video analyzers were masked meaning that the analyzers were not aware of the lubricant, and to compare these two NCTs with the contact tonometers. To compare tear film dynamics in response to contact and NCTs, subjects were tested with four tonometers.

Two NCT devices were used: Ocular Response Analyzer (ORA; Reichart Ophthalmic Instruments, Buffalo, NY, USA) and Corvis ST (Oculus, Wetzlar, Germany). The two contact tonometers used in this study included a rebound tonometer, iCare (Tiolat Oy, Helsinki, Finland) and an applanation tonometer, Tono-Pen (Reichart Ophthalmic Instruments).

To evaluate the effect of tear load from an eye drop administration on droplet formation, NCT tests were repeated with administration of one drop of lubricant immediately after the initial standard examination with NCT devices. A preservative free lubricant eye drop with a viscosity close to that of the tears was used for these tests which contained 0.1% dextran 70 and 0.3% hypomellose (GenTeal Tears; KC Pharmaceuticals Inc., Pomona, CA, USA). Each eye drop was administered by slightly tilting the subject's head back and pulling the lower eyelid downward, away from the cornea. The eye drop vial would then be held over the eyelid pocket and one drop would be gently administered. The testing order was as follows: (1) the first NCT device, (2) the first NCT device after one drop of lubricant was administered, (3) the second NCT device, (4) the second NCT device after one lubricant eye drop was administered, (5) iCare testing, and followed by (6) administration of topical anesthetic containing 0.5% proparacaine hydrochloride ophthalmic solution (Akorn, Inc., Lake Forest, IL, USA) and Tono-Pen testing. Note that several minutes elapsed between tests two and three to allow for movement of the NCTs and camera setup. Further, to account for the potential effect of order of testing, 10 subjects received ORA testing first, followed by CorVis ST, whereas the other 10 subjects received CorVis ST measurement first, followed by ORA. To evenly distribute the order between the two NCT devices, ORA and Corvis ST were selected as the first device for every other subject.

**Figure 1.**
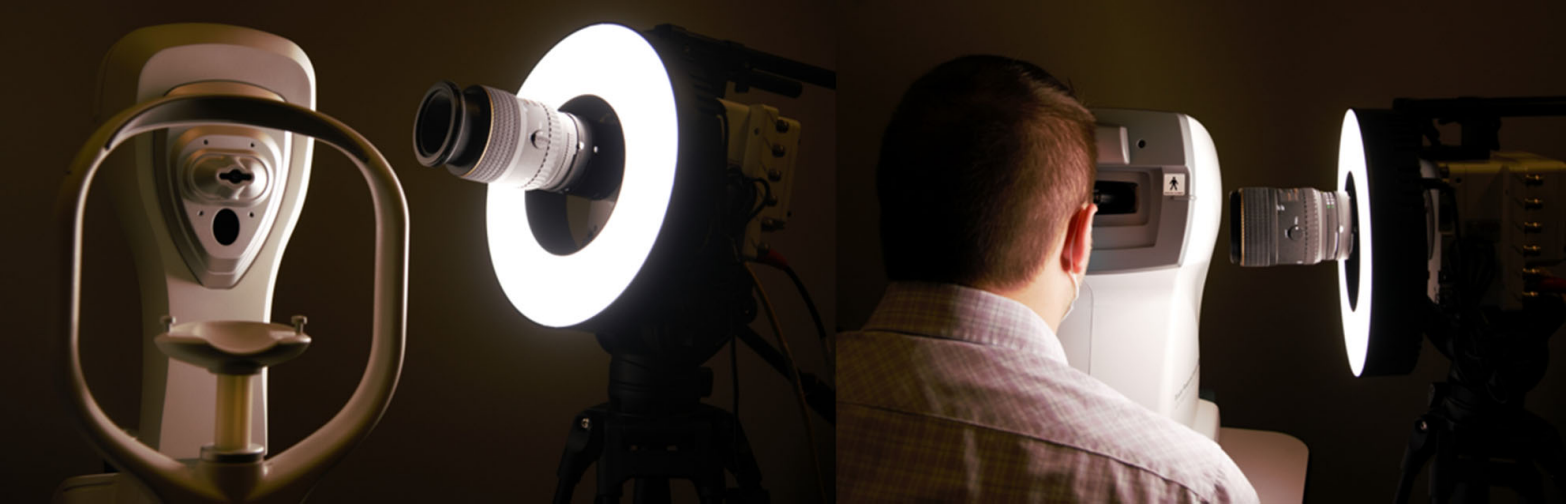
The experimental setup showing LED light mount on the camera and its location with respect to the CorVis ST and ORA devices.

A high-speed video camera (Phantom VEO 340S; Vision Research, Inc., Wayne, NJ, USA) coupled with a 100 mm f/2.8 macro lens was used to capture corneal deformation, eye lid motion, tear film dynamics, and droplet formation at a spatial resolution of 20 microns/px and a frame rate of 2400 fps. Continuous and consistent lighting was produced by a 10-inch bi-color, on-camera, LED ring light (Smith-Victor, Bartlett, IL, USA). The camera was placed perpendicular to the direction of the air puff to capture the temporal view of the right eye (Fig. 1).

### Image Processing, Droplet Characterization and Statistics

Videos obtained from each test were cropped to only include the duration of interest. Imaging windows were calibrated by measuring the number of pixels per inch of the ORA nozzle captured in the imaging window. Using the measurement toolbox in Phantom Camera Control software (Vision Research), tear droplets were characterized by measuring their diameter, trajectory, and velocity, as well as distance from the point of air impact to the lower tear meniscus/lid boundary. Data were descriptive in nature for demographics of the subjects and for the video analyses in terms of mean values ± standard deviation. The Student's *t*-test was performed to statistically compare each two groups.

## Results

Twenty subjects completed these tests, but one subject was excluded due to technical issues requiring multiple repeated NCT tests. The average age was 35.7 + 14.6 years and there were 10 women and 10 men.

### Contact Tonometry Tests

With respect to the contact tonometers, all subjects were tested using the iCare and Tono-Pen instruments. No droplet formation was observed with using either of these contact tonometry instruments.

With respect testing with NCT devices and video analyses, the results are divided into two groups based on whether the subject did or did not receive a lubricant eye drop before NCT testing. Moreover, a detailed qualitative overview of the subjects with droplet formation divided into predominantly forward motion and predominantly downward motion is provided to assess tear dynamics in response to air puff deformation.

### NCT Tests Without Eye Drop Administration

There were 38 NCT testing videos processed without eye drop administration. No droplet formation was observed in any of the videos during these standard clinical testing without an eye drop. Thirty-two videos showed no pooling of the tear film whereas the other six reflected a pooling of tears at the lower eyelid meniscus.

### NCT Tests With Lubricant Eye Drop Administration

There were 38 test videos processed using the 2 NCT devices after administration of a single drop of lubricant eye drop. Among these videos, 14 showed no pooling of tears at the meniscus and no sign of droplet formation. Twelve videos showed tears pooling at the lower tear meniscus. Eleven videos showed droplet formation, where the drops initiated from the tear meniscus/lid boundary and reflected a predominant downward motion after they departed the subjects’ eye lashes. One video reflected droplet formation where the droplets showed a predominant forward trajectory. All droplets were emitted from the inferior tear meniscus-eyelid margin, whether predominantly moving forward or downward, respectively. No droplets were launched from the corneal or scleral surfaces until an obstruction to flow was reached. The single video showing droplets with a predominantly forward trajectory was using the ORA with administration of an eye drop, and the first NCT device examination. The droplets were generated at the inferior tear meniscus/lid boundary.

### Droplet Characterization

#### Droplet Size and Trajectory

**Figure 2.**
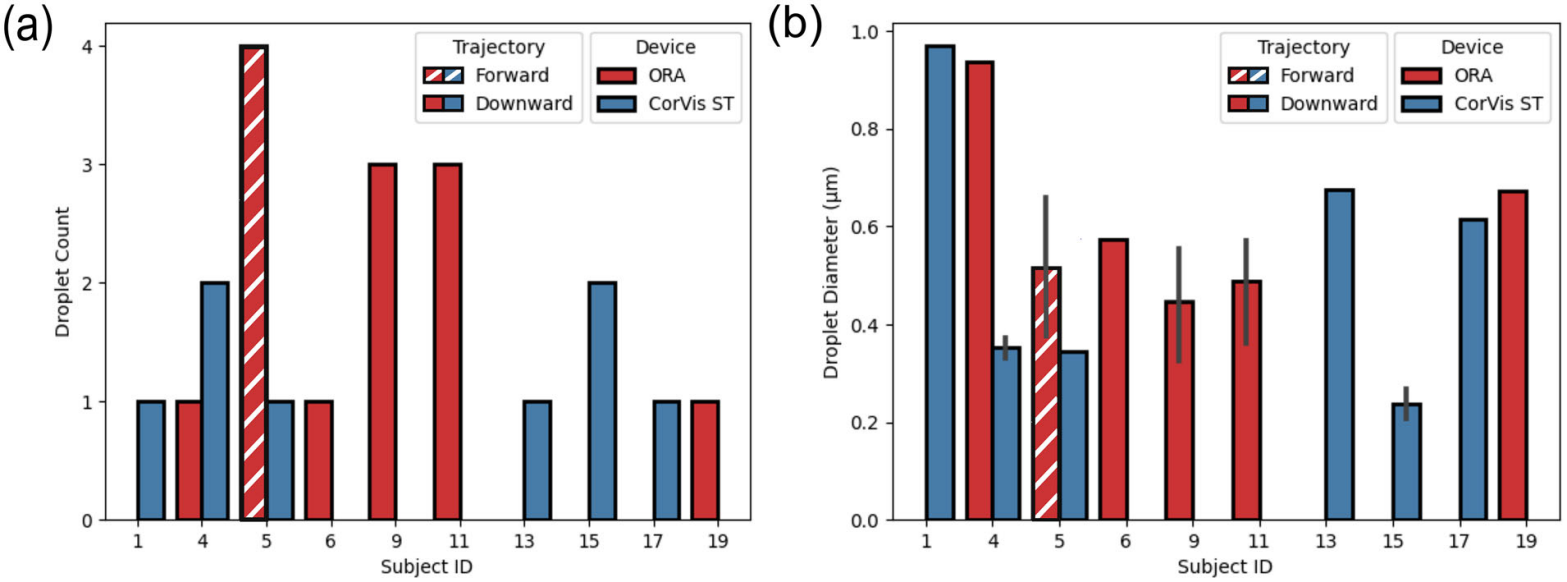
Distribution of (**a**) droplet count, and (**b**) droplet diameter, for NCT examinations CorVis ST and ORA after instillation of a lubricant eye drop. Solid colors represent downward trajectory and diagonal stripes represent forward trajectory.

Droplet diameters were measured from the 11 videos demonstrating droplets after NCT testing following administration of a lubricant eye drop. Among these 11 videos, the total number of droplets measured from all videos was 21, with 13 drops from 6 subjects using the ORA device and 8 drops from 6 subjects corresponding to the CorVis ST device. Out of the six subjects, two showed droplet formation with both devices. The droplet diameter (*n* = 21) ranged between 210 µm and 970 µm, with an average of 500 ± 200 µm. Figure 2 shows a summary of droplet count and droplet diameters based on NCT device and droplet trajectory. There was no significant difference in droplet diameters between the two NCT devices (*P* value = 0.5151). Further, no significant difference was found in the droplet size between those with forward (*n* = 4) versus downward (*n* = 17) trajectory (*P* value = 0.3605).

#### Droplet Velocity

Droplet velocity magnitude was measured for the single video that generated droplets with a predominant forward trajectory. Four droplets were identified with predominantly forward motion. Two of these stayed in focus through multiple frames, and the velocity magnitudes and angle of motion calculated for these 2 droplets were 0.93 and 0.50 m/s at 23 degrees and 70 degrees from the air puff direction axis, respectively.

#### Tear Film Response

**Figure 3.**
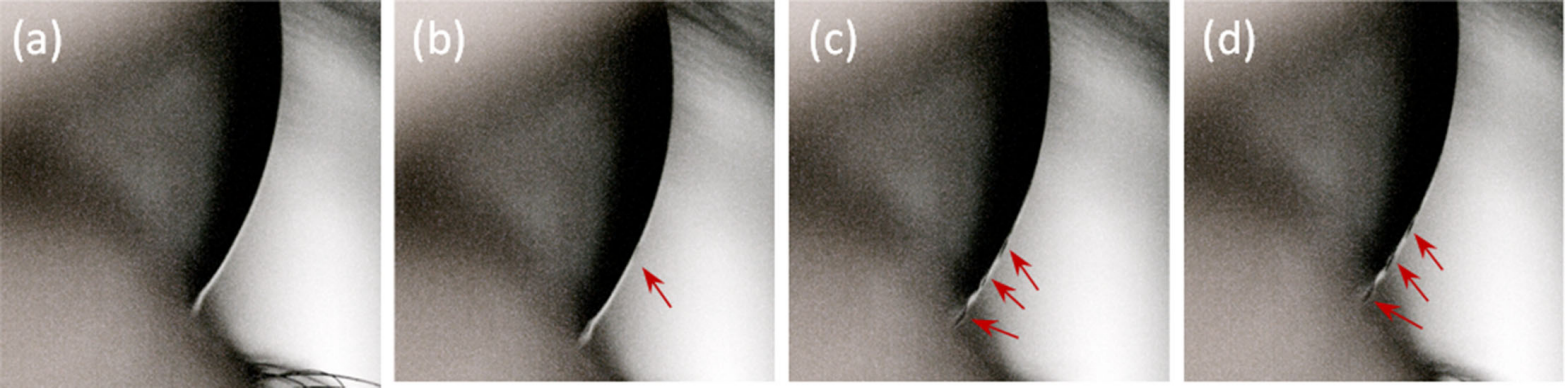
Tear wave tracking using ORA device on a subject after one eye drop is administered. Red arrows mark the tear wave, (**a** to **d**) progress in time.

Tear film hydrodynamics in response to air puff deformation were captured via high-speed imaging. Examinations without lubricant eye drop application showed pooling of the tears at the lower meniscus, whereas those with an eye drop added showed that the tear film moves away from the apex and toward the lid/eyelashes, showing similar behavior as the capillary wave in water ripple effect. This behavior was only evident when a lubricant eye drop was administered and created a larger tear load. As shown in Figure 3, which demonstrates the tear film response to the ORA air puff after an eye drop is administered, the tear film remained adhered to the cornea while moving parallel to the corneal surface, following the corneal curvature. This behavior where fluid flow tends to remain attached to the curved solid surface is explained via the Coanda effect.^16^^,^^17^ In the cases with droplet formation predominantly in the downward direction, the droplets detached from the vicinity of tear meniscus-eyelid boundary, which interrupted the tear wave, and moved through the eyelashes, launching at the tips.

To evaluate the role of the distance between air puff impact location at the apex and the obstruction to flow at the meniscus-eyelid boundary, this distance was measured for all examinations performed using the two NCT devices. The measured values were 6.12 mm ± 1.2 mm for CorVis ST examinations (*n* = 19), 5.8 mm ± 0.2 mm for ORA tests (*n* = 19). The only ORA test in which droplets launched with predominant forward motion had the shortest distance between the corneal apex and lower meniscus of 3.81 mm.

**Figure 4.**
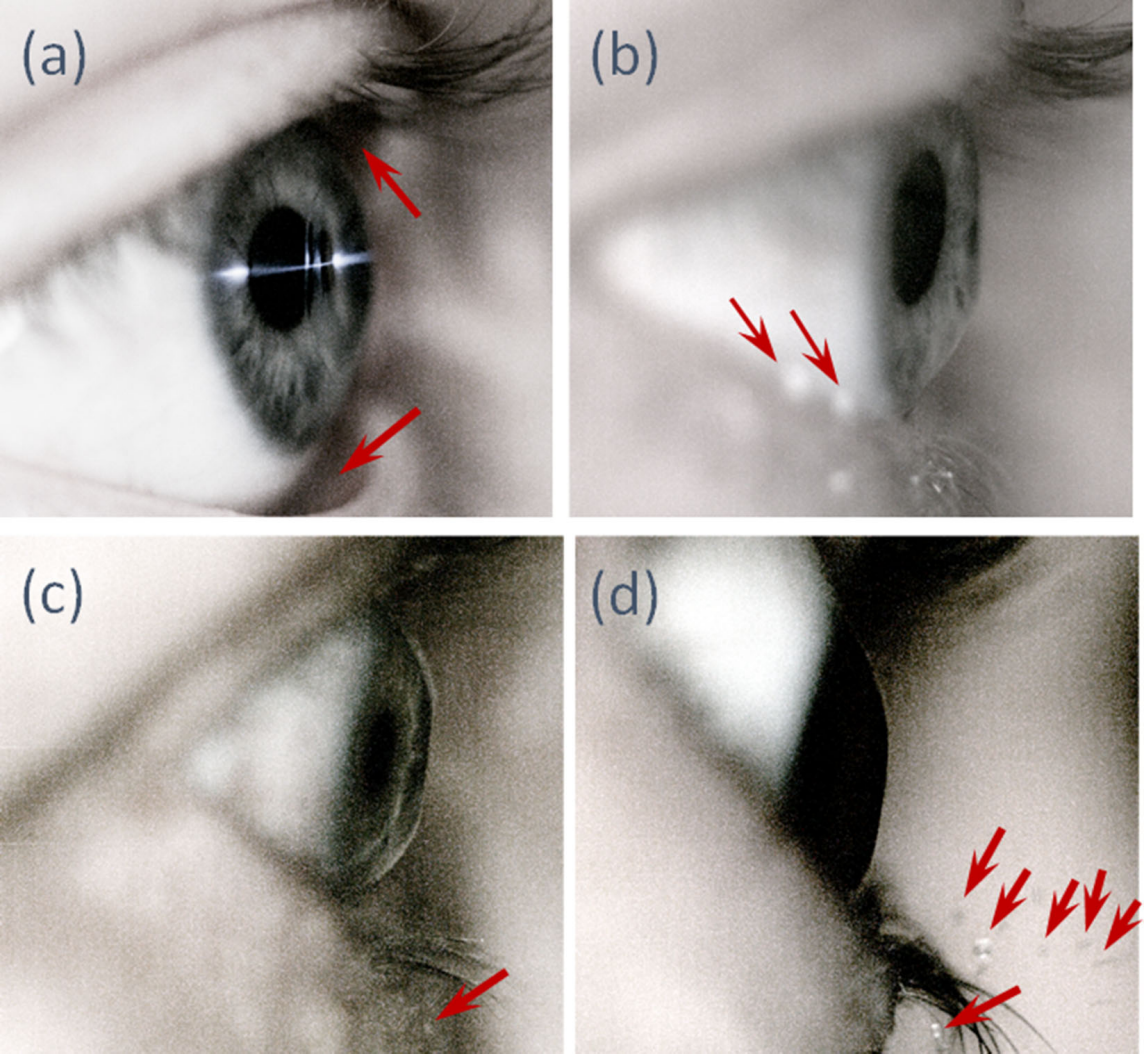
Tear meniscus and droplet trajectory captured in still images from the video. The red arrows indicate different responses of eyelid and tear film to the air puff impact. (**a**) Eyelid displacement from the globe due to the air puff impact on the cornea. (**b**) Tear drops along the inferior edge of the lower meniscus-eyelid. (**c**) droplet falling downward along the eyelashes. (**d**) droplets with predominant forward trajectory.

To summarize the dynamic interaction between air puff and the eye, we can identify five different responses, including no detectable pooling or droplet formation, eyelid separation from the globe, tear drops tracking the edge of meniscus-eyelid in the backward direction, droplet formed predominantly in the downward direction, and droplet formed with a predominantly forward trajectory (Fig. 4). Supplementary Video S1 demonstrates the eyelid separation from the globe and corneal deformation during the time frame of the air puff. Supplementary Video S2 demonstrates the droplet formation with a forward trajectory.

## Discussion

To further assess the air puff and tear film interaction, the dynamics of the air puff exerted by each of the two NCT devices was considered on the tear film. The air puff magnitude of the CorVis ST is consistent and greater than the air puff magnitude of the ORA, which is variable, depending on the IOP of the subject. Videos that captured the air puff deformation after the administration of a lubricant eye drop provided the optimal setting to study the air puff interaction with the tear film because of a larger tear load. Under this condition, the air puff arrives at the corneal surface, the tear film is pushed radially away from the apex while remaining attached to the corneal surface. This behavior, explained by the Coanda effect,^16^^,^^17^ occurs due to the tendency of the tear film to follow the ocular surface curvature during deformation and results in droplets leaving the eye only at the eyelid boundary which interrupts tear flow.

**Figure 5.**
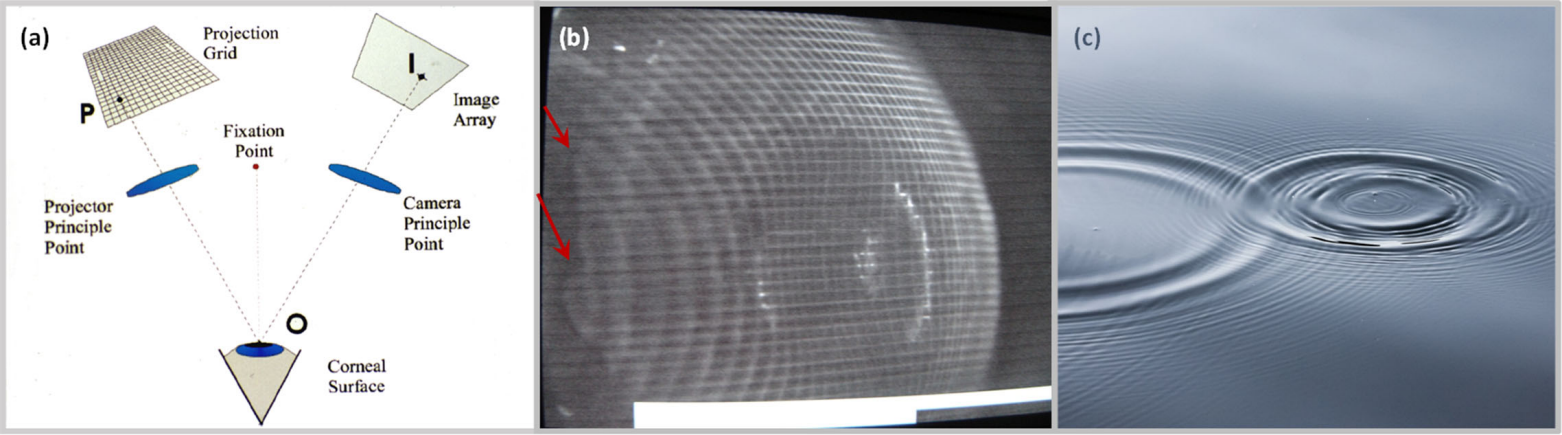
Air puff interaction with the tear film captured through fluorescein imaging. (**a**) Schematic showing device setup, showing an off-axis excitation grid projected on the eye from one side, and the fluorescent emission of the grid pattern from the anterior corneal surface imaged off axis from the other side. (**b**) Fluorescence imaging of air puff impacting human cornea demonstrating the Coanda effect in tear film, where the tear wave is shown traveling outward from area of deformation, indicated by the red arrows. (**c**) Raindrops impacting the surface of water, creating a circumferential wave, expanding in the radial direction, and ripple effect.

To better demonstrate this behavior, the tear film displacement captured using an ORA device, augmented with an integrated surface topography system to track deformation of the surface during the air puff^18^ (Fig. 5a) is shown in Figure 5b, where the air puff interaction with a subject's eye is captured with the application of a fluorescent eye drop. The edge of the tear film wave traveling radially from the center is shown with red arrows. This resembles that of the water surface impinged by a drop of rain (Fig. 5c). As the tear film moves further away from the location of the impact, the tear wave approaches an obstruction in the tear meniscus and eyelid. Depending upon the wave velocity and surface tension, the meniscus obstruction could result in droplet formation or only tear drops tracking the edge of the meniscus-eyelid boundary. The kinetic energy of the tear film displacing radially can be characterized using continuity equation. Assuming the tear film flow is incompressible, axisymmetric, flowing radially across a flat cornea, and that the film thickness does not decrease appreciably in the distance range under consideration, the kinetic energy can be simplified to:

(1) $$E_{K}=\frac{\rho }{2}2\pi v_{r}^{2}rh=\frac{c_{1}}{r}$$

As shown in Equation 1, the kinetic energy is expected to decrease as at least *1/r* where r is the distance traveled. Furthermore, as demonstrated in Equation 1, the tear wave's kinetic energy decreases as the tear wave moves further away from the impact location. Thus, smaller distances between the air puff impact location and the meniscus-eyelid boundary would leave greater kinetic energy and increase the potential of droplet formation. This equation accounts for variations between individuals with respect to orbital anatomy with relative position of the eye in a shallow versus deep setting. Specifically, in an individual who has a relatively shallow eye within the orbit, there is a short distance between meniscus-eyelid and air puff impact location which resulted in droplet formation with forward trajectory. This tear wave behavior can be explained by the higher kinetic energy at the time the tear wave encountered an obstruction at the meniscus-eyelid boundary. In other words, the risk of droplet formation with forward motion increases in an individual who has a shorter distance from the meniscus-eyelid boundary. It is worth noting that the upper lid was retracted during the testing, which resulted in a much shorter distance to the lower lid due to mechanical elevation of the periocular tissues.

In this study, lubricant eye drops were used to simulate subjects with a larger tear volume, similar to other studies with NCT devices.^12^^,^^14^ However, the other studies used not only low viscosity, but also medium and high viscosity eye drops, which might influence results. Despite this variation in eye drops, all studies showed that no droplet formation occurs without the addition of an eye drop. Our study design was robust with a side-by-side comparison of contact and noncontact tonometers and characterized the effect of larger tear load in the NCTs with the administration of a single eye drop. In addition, we were able to expand upon the findings by Shetty et al.^14^ by using two NCTs with distinct air puff strategies, as well as characterizing the air puff/tear film interaction. The distance between air puff impact location and tear meniscus/lid as an important potential cause for droplet formation with forward motion after an eye drop is administered. Although both devices use the same mechanism of air puff deformation to characterize ocular biomechanics and IOP, it should be noted that each device has distinct air puff strategies, which might affect response from the interaction with the tear film. However, we did not note differences based on magnitude of the air puff.

A major limitation of this work was that the study population here consisted of all healthy subjects. Further investigation is needed to assess droplet formation of NCT devices for subjects with symptoms of watery eyes, dry eyes, or with tear film instability. Additionally, due to the small number of subjects and only one case with droplet formation showing a forward trajectory, the threshold for air puff impact distance from meniscus-eyelid could not be determined. Lastly, given that droplet formation is a three-dimensional phenomenon, and the video recordings are only looking at a two-dimensional plane of view, the droplet diameter measurements in this study depend upon the distance in the depth of field and could render our droplet diameter measurements inaccurate. In addition, droplets less than the pixel resolution of 20 microns could not be detected.

In order to reduce the risk of droplet formation, it is recommended to avoid lubricating drops, as in previous reports using other NCT devices with various spatial and temporal profiles of the air puff. In addition, our study indicates the shorter the distance that the tear wave travels before it impacts the lid, the greater risk of launching droplets with predominantly forward motion if lubricant eye drops are used. Therefore, it is recommended to avoid holding open the upper lid alone which pulls up the lower lid and shortens the distance the tear wave travels to the lower lid. Rather, it is recommended to hold open both the upper and lower lids if the patient has difficulty maintaining open eyes for the examination. Finally, it was recently reported in symptomatic COVID-19 positive patients, only 7% showed COVID RNA in the tear film.^19^ Therefore, NCTs are unlikely to pose a risk of transmitting COVID infection in asymptomatic patients.

In conclusion, videos of the hydrodynamic interaction between air puff and tear film were carefully analyzed to characterize droplet generation. No droplets were produced in standard operation of specific NCTs investigated, or with either of the two contact tonometers. Instillation of a single eye drop results in droplet formation 31.5% of the time. Of these, only one examination resulted in droplets with predominantly forward motion, likely due to the subject holding the upper lid, resulting in an abnormally short distance from the point of air puff impact to the meniscus-eyelid boundary. Future studies are indicated for subjects with dry eye or other sources of unstable tear film.

## Supplementary Material

Supplement 1

Supplement 2

## Supplementary Material

**Supplementary Video S1**. CorVis ST examination on a subject after application of a lubricant eye drop demonstrating the reflection of corneal deformation and the eyelid separation from the globe because of air puff impacting the eye.

**Supplementary Video S2**. ORA examination on a subject after application of a lubricant eye drop demonstrating droplet formation. The droplets have a predominant forward motion.
